# In Search of a Germ Theory Equivalent for Chronic Disease

**DOI:** 10.5888/pcd9.110301

**Published:** 2012-05-10

**Authors:** Garry Egger

## Abstract

The fight against infectious disease advanced dramatically with the consolidation of the germ theory in the 19th century. This focus on a predominant cause of infections (ie, microbial pathogens) ultimately led to medical and public health advances (eg, immunization, pasteurization, antibiotics). However, the resulting declines in infections in the 20th century were matched by a rise in chronic, noncommunicable diseases, for which there is no single underlying etiology. The discovery of a form of low-grade systemic and chronic inflammation (“metaflammation”), linked to inducers (broadly termed “anthropogens”) associated with modern man-made environments and lifestyles, suggests an underlying basis for chronic disease that could provide a 21st-century equivalent of the germ theory.

## Introduction

Throughout history, infections have posed the biggest challenge to human health. This challenge changed in the 19th and 20th centuries because of economic development and improvements initiated largely by the Industrial Revolution — public health and hygiene, the advent of antibiotics and vaccinations, and, driving these, the consolidation of the germ theory of disease (1). It seemed that man’s battle against disease had been all but won.

Mid-20th–century optimism, however, was dampened by the reality of an epidemiological transition (2) that occurs with economic development. In this transition, chronic diseases and conditions (eg, heart disease, cancer, diabetes, chronic respiratory problems) — often called the “diseases of civilization” (3) — replace infections as the major source of disease. (Chronic diseases and conditions are defined here as those that are noncommunicable, lasting, recurrent, and without a primary microbial cause. These conditions can include injuries [eg, motor vehicle trauma, occupational/sports injuries] but exclude acute diseases [eg, AIDS] that have become chronic through advances in medical treatments.) The epidemiological transition occurred in the latter half of the 20th century for many developed countries; approximately 70% of diseases now result from chronic conditions (4). The same transition is occurring now in rapidly developing countries, such as China and Mexico, and is predicted in late-developing countries, such India and Bangladesh (1). Harris (1), Anderson (5), and others have charted the differences in thinking these changes have brought to modern epidemiology, emphasizing the difficulties in assigning causality when shifting from a mono-causal focus (promoted by the germ theory to address infectious disease) to a multi-causal focus to address chronic disease. 

No equivalent of the germ theory has provided a unifying understanding of chronic disease etiology. The aging of the population and the dysmetabolism associated with aging has affected the prevalence of chronic disease; however, the increase in the prevalence of chronic diseases and associated risk factors and behaviors among all age groups limits aging as a sole explanation. Genetic influences and gene–age interactions are also incomplete explanations, in light of the sudden increase in and other known causes of chronic diseases. Many behaviors and environmental factors have been implicated, but a unifying theoretical underpinning has not been identified.

The discovery of a form of otherwise unrecognized inflammation in the early 1990s (6) and its widespread presence in many chronic diseases (7) led to the suggestion that many, if not all, such diseases may have this type of inflammatory basis (8). If so, and if a unifying cause could be identified to explain what is essentially a “multi-causal enterprise” (5), the implications for the management of chronic conditions could be significant, possibly reflecting the influence of the germ theory on changes in infectious disease prevention, diagnosis, treatment, and control.

## Inflammation and Disease

For more than 2,000 years, classical inflammation has been recognized by the symptoms identified by the Roman physician Aurelius Celsus as pain (*dolor*), redness (*rubor*), heat (*calor*), and swelling (*tumor*), with the more recent addition of loss of function (*torpor*). This form of classical inflammation is typically a short-term response to infection and injury, aimed at removing the infective stimulus and allowing repair of the damaged tissue, ultimately resulting in healing and a return to homeostasis. However, in 1993, researchers discovered a different type of prolonged, dysregulated, and maladaptive inflammatory response associated with obesity, which they suggested may explain the disease-causing effects of excessive weight gain (6). “Metaflammation” (9), as it was later called because of its link with the metabolic system, differs from classical inflammation in that it 1) is low-grade, causing only a small rise in immune system markers (ie, a 4- to 6-fold increase vs a several-hundred-fold increase); 2) is persistent and results in chronic, rather than acute, allostasis; 3) has systemic rather than local effects; 4) has antigens that are less apparent as foreign agents or microbial pathogens and, hence, have been referred to as “inducers”; 5) appears to perpetuate, rather than resolve disease; and 6) is associated with a reduced, rather than increased, metabolic rate.

**Figure 1:**
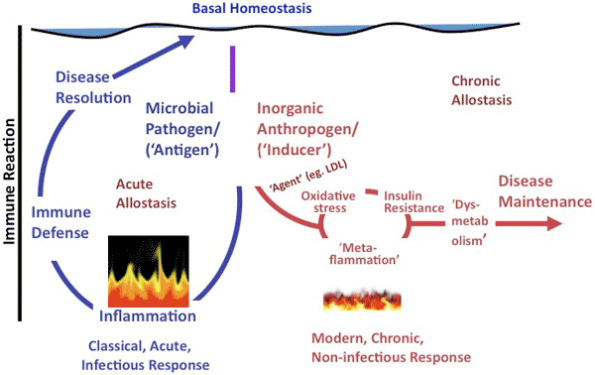
A representation of the difference between classical inflammation (illustrated as raging fire), initiated by a microbial antigen or injury, and metaflammation (illustrated as smoldering fire), caused by inorganic “anthropogens.” Adapted from Egger and Dixon (10). Abbreviation: LDL, low-density lipoprotein. The scale of difference of immune reaction between the 2 forms (ie, approximately 100-fold) is not shown.

In essence, although classical inflammation has a healing role in acute disease, metaflammation, because of its persistence, may have a mediating role, helping to aggravate and perpetuate chronic disease. The difference between these 2 forms of inflammation is illustrated in Figure 1.

Metaflammation has been associated with many chronic diseases, conditions, and risk factors, ranging from type 2 diabetes and depression to heart disease, many forms of cancer, and even dementia (7). Possible reasons for these associations include facilitation of atherosclerosis (11), development of insulin resistance (12), endoplasmic reticular stress (13), and changes in gut microbiota (14). Metaflammation may be part of a causal cascade, including endoplasmic reticular stress and insulin resistance, or simply a defensive reaction to persistent stimuli that induce chronic disease. However, mounting evidence suggests metaflammation may develop as an intermediate immune system response to certain inducers, which, if maintained, can lead to the development and maintenance of dysmetabolic conditions and chronic disease (7,9). A commonly expressed view is that obesity is a prerequisite for metaflammation to occur or that metaflammatory inducers are necessarily either nutritional overload or nutrient-based. Epidemiologic data suggest, however, that although several metaflammatory inducers are indeed associated with obesity, obesity is not a necessary precursor (10,15); it may simply represent a “canary in the coal mine,” warning of bigger problems in the broader environment (10).

## Metaflammation and Anthropogens

An increasing number of pro- or anti-inflammatory biomarkers for measuring metaflammation have recently been identified (16), and several avenues of research have sought to identify their nutritional, behavioral, and environmental inducers (10,15). Many inducers have been identified (Table). These include not only behaviors linked to modern lifestyles facilitated by postindustrial environments — for example, poor nutrition, inactivity, inadequate sleep, and stress (17) — but also components of these environments themselves, such as particulate matter (18) and traffic-related air pollution (19); endocrine-disrupting chemicals (EDCs), called *obesogens* because of their possible link to obesity (20); social and economic conditions that create inequality and economic insecurity, including perceived organizational injustice or prejudice in the workplace (21); and the links between inequality and race/ethnicity as proposed in the “weathering hypothesis,” which suggests that childbirth outcomes are more deleterious in older mothers of certain disadvantaged racial/ethnic groups (22). Such lifestyles and environments make up a category of inducers that can be labeled “anthropogens” because of their made-made origins and potential influence on health. Anthropogens are defined here as man-made environments, their by-products, and/or lifestyles encouraged by those environments, some of which have biological effects that may be detrimental to human health.

**Table:** Pro- and Anti-inflammatory “Inducers” of Metaflammation

Evidence Level	Pro-Inflammatory (“Anthropogens”)^a^	Anti-Inflammatory (or Neutral)
**Strong**	Aging Exercise, too little (inactivity) Nutrition Excessive energy intake Fat intake saturated trans fatty acids high-fat diet Obesity/weight gain Particulate matter Smoking Sleep deprivation Stress/anxiety/depression/“burnout”	Exercise/physical activity/fitness Intensive lifestyle change Nutrition Restricted energy intake Fish/fish oils Fruits/vegetables Nuts Weight loss
**Moderate**	Nutrition Fast food/Western-style diet High omega 6:omega 3 ratio Fiber (low intake) Fructose Glucose High-glucose/glycemic-index foods High glycemic load Glycemic status Air pollution (indoor/outdoor) Inequality/economic insecurity	Nutrition Alcohol (moderate intake) Capsaicin Cocoa/chocolate (dark) Fiber (high intake) Garlic Grapes/raisins Herbs and spices Low omega 6: omega 3 ratio Mediterranean diet Olive oil Tea/green tea Vinegar Smoking cessation
**Limited**	Exercise, excessive Nutrition Starvation Alcohol (excessive/bingeing) Meat (domesticated) Sugar-sweetened drinks Endocrine disrupting chemicals Low perceived workplace fairness “Sick building syndrome” Secondhand smoke Thermal comfort (eg, air conditioning) Low socioeconomic status	Nutrition Breast milk Dairy calcium Eggs Lean game meats Low-glycemic-index foods Monounsaturated fats Soy protein

a Pro-inflammatory inducers are typically man-made, lifestyle- and/or environment-related and have been labeled *anthropogens* (10).

The underlying factors distinguishing pro-inflammatory and anti-inflammatory inducers (or neutral, in the case where there are similar pro-inflammatory inducers) appear to be both temporal and of human origin (Table). All anti-inflammatory or neutral inducers are those with which humans have had experience over hundreds or thousands of years, most of which are natural (eg, fruits, nuts) or minimally modified (eg, wine, beer). Pro-inflammatory inducers are recent and man-made (eg, EDCs), are modified (eg, processed food) or induced (eg, inactive lifestyle), or are outcome effects (eg, income/social inequality) of a man-made environment. Aging is an interesting pro-inflammatory inducer. The typical dysregulatory and metaflammatory effects of aging (also called *inflammaging* [23]) are reduced by healthy lifestyles. Hence, aging, although immutable, can be considered in the scope of an anthropogen, as the increase in longevity in modern populations can be ascribed to positive anthropogenic factors (eg, medicine, immunization), leading to the decline of the infections.

**Figure 2:**
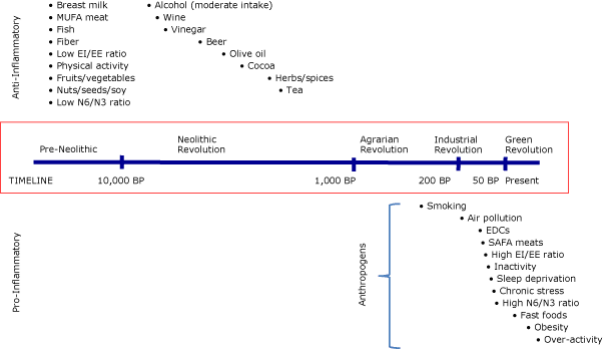
The pro- or anti-inflammatory effects of various inducers and their approximate (not to scale) introduction into the human environment. The bullets associated with each inducer in the time frame indicated suggest the approximate time of introduction to the human environment. “Anthropogens” are defined here as man-made environments and the by-products, behaviors, and/or lifestyles encouraged by those environments, some of which have biological effects which may be detrimental to human health. Abbreviations: MUFA, monounsaturated fatty acid; EI, energy intake; EE, energy expenditure; N6, omega-6 fatty acid; N3, omega-3 fatty acid; BP, before present; EDCs, endocrine-disrupting chemicals; SAFA, saturated fatty acid.

Pro- and anti-inflammatory inducers are related to major changes in human evolution (Figure 2). The split between anti-inflammatory or neutral inducers and pro-inflammatory inducers (anthropogens) is based on time and the amount of human involvement in developing such inducers (eg, food processing, time-saving machinery).

## Chronic Disease and the Germ Theory

Given our modern understanding of the immune system, the ways in which anthropogens affect it could be revealing. Human immune responses are either innate or developed through exposure to unfamiliar stimuli over an extended time. Nontoxic antigens with which humans have evolved over thousands of years (“good germs”) are likely to cause little immune response, whereas anthropogens — man-made, novel, and recently introduced — are more likely to cause a response, albeit a low-level one, in a not-immediately-life-threatening situation. If exposure persists, the response may become chronic. A discussion of the possible mechanisms for this response is beyond the scope of this article, but the mechanisms may be related to genetic or epigenetic influences on chemical receptors, such as through nutritional factors (24). Because such a response is undifferentiated, it is likely to be systemic rather than localized.

What does the immune response tell us about the inducers identified in this article? It seems the human immune system is reacting to new, man-made environments and lifestyles (anthropogens), to which it has been exposed for a brief period (ie, since the beginning of the Industrial Revolution, approximately 1800 CE) and to which the immune system has not had time to develop. To characterize this reaction as lifestyle-based offers only part of the explanation. For the last 30 to 40 years, in particular, we have experienced the emergence of a sedentary, nutritionally engineered, consumption-driven, security-conscious, screen-focused environment, the likes of which have never before been experienced by humans. The fact that most chronic diseases have also risen to prominence during this time (albeit along with an aging population and its genetic propensities) would point to the modern industrial and postindustrial environment as a distal “cause of the cause” of modern chronic diseases.

These ideas are not meant to imply that such a modern-day environment has not benefitted human health. It has been primarily responsible for the decline in infectious diseases during the last 1 or 2 centuries. The rise in chronic diseases is a recent aberration and probably reflects an inevitable point of diminishing returns in a system of exponential economic growth, which is being reflected in our physiologic systems. Anthropogen-induced metaflammation could offer a generic etiology for chronic diseases of similar import, albeit slightly different in scope, to that of the germ theory for infectious diseases.

## Relevance for Public Health

The germ theory had immediate implications for clinical medicine. Its relevance was extended to public health through the concept of the epidemiological triad, which stressed the need for attention to all 3 corners of a triangle: host, vector (and agent), and environment, for effective control of disease outbreaks. A focus on germs (ie, agents) alone was insufficient. John Snow’s removal of the handle of the Broad Street pump during the London cholera epidemic of the 1850s showed the relevance of both vector and environment to infection control.

Although less frequently applied, the triad has also proved robust in its application to chronic disease. The concept of anthropogens, as proposed here, offers a broader base for managing chronic disease than the germ theory did for infectious diseases. It offers a single concept for 2 corners of the triad, environment and vector (and agent). Although agents can be inducers of metaflammation — either exogenous or endogenous (eg, EDCs, excessive cortisol levels) — these agents, plus the vectors through which they are delivered (eg, polluted air/water, socioeconomic stress) and environments in which they exist (eg, industrial output, inequality) are included under the single concept of disease-promoting anthropogens. Public health approaches in this model thus become generic, rather than fragmented (eg, dietary intakes rather than specific nutrients), and distal, rather than proximal (eg, industrialization/economic growth rather than fast-food marketing). Most important, such approaches need to involve disciplines often otherwise not considered in discussions of epidemic illness — such as macroeconomics, geography, ecology, and even business — in considering big-picture causality.

## Managing Anthropogens

Obviously, not all anthropogens are unhealthy (although there are many others, not listed here, that possibly are [eg, asbestos in building materials, shift work, increased atmospheric carbon dioxide]). Any suggestion of halting human progress or returning to Paleolithic conditions would be akin to trying to wipe out all germs — good and bad — to manage infections. Acknowledgment of unhealthy anthropogens, however, would shift the focus from organic and multifocal causes of chronic diseases to vectors and environments and their distal economic, social, and physical drivers, just as the germ theory focused on microbial pathogens. This approach would also deflect attention from a simple lifestyle-based explanation. A focus only on lifestyle could inadvertently deflect criticism from systemic causes and put blame on the individual, whereas the recognition of an anthropogen-based etiology would cast a wider net.

The applications of this approach reside in the refocusing of testable hypotheses associated with chronic disease causality, as well as the recognition of a wider scope of influences on modern health and well-being that may incorporate a spectrum of current world issues, such as population growth and climate change (25). Pharmaceutical, medical, and surgical treatments will undoubtedly remain crucial in the treatment of chronic disease, as they are with infections, but an anthropogen-based etiology suggests the need for a bigger perspective on disease prevention and management. Once this perspective is accepted, we can make prevention our primary endeavor. Whatever it is that our immune system is telling us, perhaps it is time to listen.

## References

[1] Harris B . Public health, nutrition and the decline of mortality: the McKeown thesis revisited. Soc Hist Med 2004;17:379-407.

[2] Sanders JW , Fuhrer GS , Johnson MD , Riddle MS . The epidemiological transition: the current status of infectious diseases in the developed world versus the developing world. Sci Prog 2008;91(Pt 1):1-37. 10.3184/003685008X284628 18453281 PMC10367498

[3] Björntorp P . Visceral obesity: a “civilization syndrome.” Obes Res 1993;1(3):206-22. 16350574 10.1002/j.1550-8528.1993.tb00614.x

[4] Lopez AD , Mathers CD , Ezzatti M , Jamison DT , Murray CJ . Global and regional burden of disease and risk factors, 2001: systematic analysis of population health data. Lancet 2006;367(9524):1747-57. 10.1016/S0140-6736(06)68770-9 16731270

[5] Anderson H . History and philosophy of modern epidemiology. http://philsci-archive.pitt.edu/id/eprint/4159. Accessed October 16, 2011.

[6] Hotamisligil GS , Shargill NS , Spiegelman BM . Adipose expression of tumor necrosis factor-alpha: direct role in obesity-linked insulin resistance. Science 1993;259(5091):87-91. 10.1126/science.7678183 7678183

[7] Libby P . Inflammation and disease. Nutr Rev 2007;65(12 Pt 2):S140-6. 10.1301/nr.2007.dec.S140-S146 18240538

[8] Scrivo R , Vasile M , Bartosiewicz I , Valesini G . Inflammation as “common soil” of the multifactorial diseases. Autoimmun Rev 2011;10(7):369-74. 10.1016/j.autrev.2010.12.006 21195808

[9] Hotamisligil GS . Inflammation and metabolic disease. Nature 2006;444(7121):860-7. 10.1038/nature05485 17167474

[10] Egger G , Dixon J . Should obesity be the main game? Or do we need an environmental makeover to combat the inflammatory and chronic disease epidemics? Obes Rev 2009;10(2):237-49. 10.1111/j.1467-789X.2008.00542.x 19055538

[11] Mizuno Y , Jacob RF , Mason RP . Inflammation and the development of atherosclerosis. J Atheroscler Thromb 2011;18(5):351-8. 10.5551/jat.7591 21427505

[12] Fernández-Real JM , Ricart W . Insulin resistance and chronic cardiovascular inflammatory syndrome. Endocr Rev 2003;24(3):278-301. 10.1210/er.2002-0010 12788800

[13] Hotamisligil GS . Endoplasmic reticulum stress and the inflammatory basis of metabolic disease. Cell 2010;140(6):900-17. 10.1016/j.cell.2010.02.034 20303879 PMC2887297

[14] Bäckhed F . 99th Dahlem Conference on Infection, Inflammation and Chronic Inflammatory Disorders: the normal gut microbiota in health and disease. Clin Exp Immunol 2010;160(1):80-4. 10.1111/j.1365-2249.2010.04123.x 20415855 PMC2841839

[15] Wärnberg J , Nova E , Romeo J , Moreno LA , Sjostrom M , Marcos A . Lifestyle-related determinants of inflammation in adolescence. Br J Nutr 2007;98 Suppl 1:S116-20. 10.1017/S0007114507839614 17922948

[16] Ouchi N , Parker JL , Lugus JJ , Walsh K . Adipokines in inflammation and metabolic disease. Nat Rev Immunol 2011;11(2):85-97. 10.1038/nri2921 21252989 PMC3518031

[17] Willett WC , Koplan JP , Nugent R , Dusenbury C , Puska P , Gaziano TA . Prevention of chronic disease by means of diet and lifestyle changes. In: Jamison DT, Breman JG, Measham AR, Alleyne G, Claeson M, Evans DB, et al, editors. Disease control priorities in developing countries. 2nd edition. Washington (DC): World Bank; 2006. 21250366

[18] Mazzoli-Rocha F , Fernandes S , Einicker-Lamas M , Zin WA . Roles of oxidative stress in signalling and inflammation induced by particulate matter. Cell Biol Toxicol 2010;26(5):481-98. 10.1007/s10565-010-9158-2 20340042

[19] Alexeeff SE , Coull BA , Gryparis A , Suh H , Sparrow D , Vokonas PS , Medium-term exposure to traffic-related air pollution and markers of inflammation and endothelial function. Environ Health Perspect 2011;119(4):481-6. 10.1289/ehp.1002560 21349799 PMC3080929

[20] Neel BA , Sargis RM . The paradox of progress: environmental disruption of metabolism and the diabetes epidemic. Diabetes 2011;60(7):1838-48. 10.2337/db11-0153 21709279 PMC3121438

[21] Elovainio M , Ferrie JE , Gimeno D , Devogli R , Shipley M , Vahtera J , Organizational justice and markers of inflammation: The Whitehall II study. Occup Environ Med 2010;67(2):78-83. 10.1136/oem.2008.044917 19773285

[22] Geronimus AT . The weathering hypothesis and the health of African-American women and infants: evidence and speculations. Ethn Dis 1992;2(3):207-21. 1467758

[23] Franceschi C . Inflammaging as a major characteristic of old people: can it be prevented or cured? Nutr Rev 2007;65(12 Pt 2):S173-6. 10.1301/nr.2007.dec.S173-S176 18240544

[24] Choi SW , Friso S . Epigenetics: a new bridge between nutrition and health. Adv Nutr 2010;1(1):8-16. 22043447 10.3945/an.110.1004 PMC3042783

[25] Egger G . Dousing our inflammatory environment(s): is personal carbon trading an option for reducing obesity — and climate change? Obes Rev 2008;9(5):456-63. 10.1111/j.1467-789X.2008.00469.x 18282177

